# Chronic, but not acute, fatigue predicts self-reported attentional driving errors in mothers attending infant children

**DOI:** 10.1038/s41598-019-49223-9

**Published:** 2019-09-04

**Authors:** Mar Sánchez-García, Pedro M. Valero-Mora, Eva Carvajal, Jaime Sanmartín

**Affiliations:** 1 Facultad de Psicología, Departamento de Psicología Evolutiva y de la Educación, Universitat de València, Valencia, Spain; 2 Hospital Casa de Salud Valencia, Valencia, Spain

**Keywords:** Risk factors, Fatigue

## Abstract

Mothers attending infant children usually experience high levels of fatigue, and fatigue has been shown to be related to car crashes through attentional errors, among other causes. The current study investigates the effects of fatigue on the attentional errors while driving of women attending infant children. A sample of 112 women—67 attending infant children and 45 not attending—filled out self-report questionnaires assessing acute fatigue, chronic fatigue, and attention-related driving errors. A mediational analysis showed that women attending infant children had higher levels of fatigue, and that chronic fatigue, but not acute fatigue, was related to attentional errors while driving.

## Introduction

Fatigue is one of the most commonly cited causes of car crashes, although its impact is hard to assess due to the vague terminology used, its over-involvement in severe and fatal car crashes, and the difficulty of evaluating it in such cases. So, estimations of fatalities due to fatigue vary considerably, ranging from as low as 1–4 percent to as high as 30–40 percent^1^. Despite the variations in estimations, there is consensus that it is a highly significant problem and a number of legislative counter-measures have been implemented in many countries that address the so- called risk groups−drivers that suffer drowsiness or fatigue due to the lack of sleep, the alteration of circadian rhythms or the length of time on duty. The risk groups most frequently mentioned are professional drivers, shift-turn workers, and drivers with some kind of sleep alteration, such as respiratory apnoea or narcolepsy.

A possible risk group that has not yet been studied thoroughly in relation to driving is that of mothers taking care of infant children. These women usually show symptoms of fatigue caused by changes in their everyday life^2–4^. These changes can be very disturbing, as illustrated for example by^5^, which showed that mothers with babies usually feel overwhelmed by the extra workload associated with taking care of the child when added to their other daily responsibilities.

It is reasonable to think that this fatigue may increase the risk of having car crashes. So, although research into the driving risks borne by women attending infant children is quite brief and only four studies have been identified^6–9^ they concur in pointing out the existence of such a risk. However, the scarcity of studies leaves many questions related to this topic still unanswered.

One aspect that remains to be explored is the specific mechanism that would make driving more dangerous for the women, although two findings of the study carried out by^6^ suggest some possible paths to explore: (1) mothers attending infant children often experienced driving as a sort of automatic process, so that they had no recall of their interaction with the road environment after their trips and (2) fatigue was more than just sleepiness as naps during the day only managed to alleviate the immediate feeling of sleepiness but not the overall feeling of fatigue. As it happens, these two elements point to the hypothesis that mothers attending infant children experience chronic fatigue that makes their driving automatic and prone to errors occurring during highly practiced routine activities as a consequence of lapses in attention.

The rest of the introduction will elaborate the different parts of this hypothesis, namely, fatigue, attention related cognitive errors, and their interrelationship.

## Fatigue

Fatigue has been defined as “the subjective report of a sustained sense of exhaustion with reduced motivation and capacity for physical and or mental activity”^10^. Note that this feeling of exhaustion may cause sleepiness in some cases, but it is not always so. In addition, falling asleep can be observed objectively, whereas there are no direct methods for measuring fatigue^11^. So, although they are sometimes conflated, fatigue and sleepiness have different implications in terms of diagnosis and treatment, as they are, in fact, distinct phenomena^11^.

Two types of fatigue can be distinguished: acute and chronic. This distinction is not usually discussed in the context of driving although it has important medical and psychiatric significance. Thus, acute fatigue occurs in healthy individuals, has a protective function, starts rapidly, is of short duration, and is due to a specific cause. Resting generally alleviates acute fatigue. Chronic fatigue is associated with clinically disordered populations, is perceived as abnormal, unusual, or excessive, starts insidiously, persists over time, and is usually multifactorial. It generally does not disappear after resting or via similar restorative techniques and negatively affects the daily activities and quality of life of individuals^11^.

Mothers attending infant children usually experience chronic fatigue. So^12^, mentions that “although women often believe that postpartum fatigue is a temporary condition that will lessen when everyday routines are re-established”^13^, empirical evidence suggests the contrary as, in general, postpartum fatigue does not significantly improve over the first 6 weeks after delivery^14^. Instead, postpartum fatigue either worsens progressively through the fourth week after delivery or remains stable at levels noted shortly after it^15,16^. Moreover,^17^ noted that “postpartum fatigue may continue into the second year after delivery in more than half of women”. Another additional risk factor is that postpartum fatigue has been found to be a predictor of postpartum depression^15^.

The chronic condition of fatigue in women with infant children makes their driving risks different from those of other drivers’ risk groups. So, while the job regulations of professional drivers or of shift-turn workers guarantee that they can be on duty for a certain length of time before they have the chance of getting enough rest, mothers taking care of infant children often do not have the option of having a certain number of scheduled off-duty breaks per week. In contrast, when drivers in risk groups feel fatigued, they can generally recover swiftly by getting some sleep.

As indicated by Shen *et al*.^11^ there is no scarcity of self-report scales for measuring fatigue, with more than 30 scales available for this purpose. Of these, however, we found that the Fatigue Assessment Scale (FAS)^18^ was the only one that specifically focused on measuring chronic fatigue, so we selected it for our study. We chose the Visual Analogue Scale to Evaluate Fatigue Severity (VAS-F) from all the other scales measuring acute fatigue, taking into account the advantages discussed by^19^.

## Attention Related Cognitive Errors

^20^studied the errors that tend to occur during highly practiced routine activities as a consequence of lapses in attention. These lapses are clearly a part of everyone’s life and, although they are usually harmless, they may be the cause of serious accidents too. So, the Attention-Related Cognitive Errors Scale—ARCES— was designed to measure the performance failures arising from brief failures of sustained attention. According to its authors, this scale focuses on “failures associated with inadequate monitoring of highly practiced, familiar, repetitive or tedious tasks for which there are obvious, appropriate, and adequate rules known”. As can be seen, driving falls squarely within this definition.

In a further development^21^, proposed a scale for the purposes of specifically measuring attention related errors while driving: the attention related *driving* errors scale—ARDES—, which was comprised of 19 items referring to non-intentional driving errors, such as “When I head toward a known place, I drive past it because I am not paying attention” and “On approaching an intersection, I miss a car coming down the road because I am not paying attention”.

It has been found that there is a common underlying factor in all the items with high internal consistency^21,22^ in this scale. Furthermore, in a study using an experimental paradigm (the Attention Network Test for Interactions), drivers with high ARDES scores had less processing speed and were not as well prepared to attend to high-priority signals^23^ as those with low scores. The ARDES scale has been validated in different countries^22,24^.

A concept closely related to inattention is distraction, which has been defined as “a subset of inattention, referring to all instances when attention is misallocated, but excluding cases when attention is not allocated at all.”^25^. So, an example of distraction is reading the news on the smartphone— behavior that can be measured objectively. On the other hand, inattention would equate to mind-wandering, which is harder to observe objectively than the previous example. Possibly as a consequence of that, whereas several objective distraction measures have been proposed^25^, only subjective scales are available for measuring attentional cognitive errors— although some promising developments have been explored^26^. Note that subjective scales permit a wider range of real life situations to be covered than would be easy or possible to reproduce in the laboratory, or to observe in naturalistic conditions within a short span of time.

## The Relationship Between Fatigue and Attentional Driving Errors

Understanding the way in which fatigue affects driving may help to design measures to reduce its negative effects. So, an alteration in the process of attention is frequently mentioned as one of the most critical consequences of fatigue. In particular, it is known that driving requires that attention be managed as a function of the difficulty of the task− reducing it during non-demanding periods to save resources, and increasing it during more demanding periods to circumvent hazards. However, fatigue alters how the effort is adapted to the demands of the task, with the consequence that the fatigued driver does not apply the right amount of attention needed for the particular conditions of the road^27^. This phenomenon is what seems to occur to mothers with infant children who often report feelings of driving in automatic^7^ or—as a mother participating in our study indicated— “zombie” mode. In turn, this alteration of the driver’s normal attention may lead to driving errors^21^ due to a lack of awareness of critical information−such as pedestrians crossing the road, traffic signals, or vehicle deceleration^28^.

## Goal of the Study and Hypothesis

We have seen that there is evidence that fatigue affects driving in the case of women with infants. However, little is known about whether such fatigue causes actual errors while driving. As it is known that fatigue alters attention and attention management, it seems interesting to study whether this subpopulation makes errors due to inattention. It would be important to know this in order to design countermeasures, such as those already implemented for other populations considered to be at risk (e.g. shift workers). The purpose of this study is to help fill such an information gap.

The main objective of this study is to analyse mothers attending infant children and the effects of their fatigue on attentional errors while driving. In particular, we hypothesise that these women will experience higher levels of fatigue than women not attending infant children and that, in turn, their fatigue—especially the chronic kind—will predict a higher number of attentional errors while driving.

## Design and Method

The design of this study fits the description in^29^ of a post-test only quasi-experimental study. Two groups (one treatment and one control) took part in the study, which was retrospective and survey-based. The study consisted of obtaining the responses of two groups of women to the questionnaires described in the measures section. Before the procedure started, written consent for the aggregated use of the results for research purposes was obtained from all the subjects. The methods in this study were carried out in accordance with the guidelines provided by the University of Valencia, and the protocols were approved by the Ethical Committee of the aforementioned university. As the data collection was carried out as part of a larger study^30^ that involved other tests and procedures, the participants were individually summoned to a quiet room set in our facilities, where they were provided with the forms to fill out and pencils. One of the authors of the paper was present at the time to assist the participants and to guarantee they could work without interruptions−as some of them brought their babies to the laboratory.

No extra information about the goals of the study was given to the participants before they recorded their responses. Once they had finished, the purpose of the study was explained to them succinctly and they were paid for their participation in it (30€).

### Participants

Women attending infant children and willing to participate in a study on driving and fatigue were contacted primarily in a paediatrician’s waiting room−one of the co-authors of this paper. The requirements were that they were between 25 and 50 years old, that they did not have any serious health conditions, and that they drove quite frequently. Also, their children had to be between 1 and 24 months old—as this is the period when a toddler’s sleep pattern is not yet completely established.

Women not attending infant children were contacted first at the paediatrician’s waiting room but also through the friends, relatives, and work partners of the women that had already participated in the study. The requirements for the former were the same as for the latter group, except that they had to be childless or their children had to be at least 6 years old.

The total number of participants in the study was 116, of whom 69 fulfilled the criteria for being classified as attending infant children at that moment. We will refer to this sample from now on as WAI (for Women attending infants). In turn, the other 47 fulfilled the criteria for being classified as not attending infant children and we will refer to them as WNAI (for women not attending infants).

### Measures

The variables to measure in this study were self-reports related to fatigue and to attention-related errors while driving. Below, we will discuss how these two variables were measured. Additionally, the demographic and driving variables of the participants were evaluated using several questions.

#### Fatigue

Below is a short description of the two self-report scales used in the study:

- FAS: This scale has 10 statements that refer to how often the subject feels. Each statement can be scored from one to five. Eight of the items measure fatigue directly (e.g. I am bothered by fatigue), but there are also two inverted items (e.g. I have enough energy for everyday life). It has two subscales: one related to the physical symptoms of fatigue and the other to the mental symptoms—although, for this study, they were not analysed separately and were combined in one general measure. The scale is self-administered and takes about 5 minutes to complete. The authors found a Cronbach’s alpha of 0.90. This scale has been used in several studies related to fatigue in mothers, namely^31,32^, and^33^.
- VAS-F: This scale has 18 items related to the feelings of fatigue at the time of responding. The respondents must mark each item with a circle or an X on an analogic visual scale. The scale has two subscales, namely: fatigue (13 items) and energy (5 items). Each item of this instrument consists of two opposing statements about how the subject felt at the time of responding (e.g. Indicate how you are feeling right now: Not at all tired____Extremely tired). The two statements were connected with a 100 mm horizontal line that the subject used for responding. Finally, the authors report a Cronbach’s α of between 0.91 and 0.96 for two groups of subjects—patients v. normal—and two moments of the day—morning and evening. As mentioned before, one plus of these scales is that they have been used profusely in studies into maternal fatigue^34^.

#### Attention related errors while driving

- ARDES was comprised of 19 items referring to non-intentional driving errors such as “When I head toward a known place, I drive past it because I am not paying attention” and “On approaching an intersection, I miss a car coming down the road because I am not paying attention”. The subjects employed a 5-point scale to respond to statements concerning the frequency with which the described situations happened to them, ranging from *never* or *almost never* (1) to *always* or *almost always* (5). The authors reported a Cronbach’s alpha of 0.88 and a close correlation with other validation measures, including ARCES, MAAS, dissociation^35^, and BPS. This scale has been used in^36^, and^37^.

#### Demographic and driving variables

In addition to the variables mentioned above, we also asked participants about demographic or driving issues that might potentially confound the relationship between the variables of fatigue and attention related errors. The questions were:

- Demographic: Age, currently employment status, whether the participant had a significant health problem, if she was married or similar, and number of children.
- Driving variables: Kilometres driven per week, time holding a driving licence (less than 1 year, between 2 and 5 years, more than 5 years), currently driving, whether they had had a driving incident and severity of the incident (slight, moderate, severe), and whether they had had a driving incident since the child was born and severity of such an incident (slight, moderate, severe).

### Data Analysis

The goal of our analysis is to test a model in which the fatigue, chronic or acute, of women attending an infant predicts attentional errors while driving. This type of model can be stated as a mediation model in which the condition of the women (WAI or WNAI) would be the cause, the attentional errors the outcome, and the fatigue the mediator, i.e., the mechanism through which the cause influences the consequence^38^.

Testing a mediation model involves five path coefficients; c, the total effect of the cause on the outcome; a, the effect of the cause on the mediator; b, the effect of the mediator on the outcome; c’, the direct effect of the cause on the outcome; and a*b, the indirect effect of the cause on the outcome through the mediator. Classical mediation analysis usually involves testing the four steps discussed by^39^ which requires evaluating all these coefficients under different conditions, but current practice^38^ (p. 113–119) relies only on evaluating whether the indirect effect is other than zero. Finally, although the actual testing of the coefficient can be carried out using regression, software for structural equation models is often used for that purpose as it not only permits the analyses to be performed in one step but also allows for the inclusion of latent variables if needed^40^. So, in our case, data were managed and analysed with SPSS 24 and the library lavaan^41^ in R^42^.

## Results

Table 1 (numerical variables) and Table 2 (categorical variables) show descriptive statistics for the samples of women participating in the study. The age of the participants in both groups was very similar, but women not attending infants drove an average of 68.8 km more than those attending infants, although this difference was not statistically significant. There were similar percentages of mothers with jobs in both groups,. As expected, the women attending infants were more often involved in a relationship than the other group of women. Only one subject manifested that she suffered from some health-related problems, but they were not sufficiently severe to affect her driving and she was not excluded from the study. None of the subjects had a job involving work shifts. More women not attending infants than attending (23.3% versus 9%) indicated that they had had at least one traffic incident in the past. Finally, three women attending infants had suffered accidents of slight severity since their children had been born, and another three had had accidents of moderate severity.

**Table 1:** Age and kilometres per week of the mothers attending infants (WAI) and not attending infants (WNAI).

		N	Mean	St. Dev.	Median	Min.	Max.	Range	Skew	Kurtosis	Std. Err.	diff	t	p
Age	WAI	67	34.6	5.09	35	23	51	28.00	0.17	0.47	0.63			
	WNAI	45	36.9	7.94	36	25	53	28.00	0.18	−1.08	1.18	−2.3	−2	0.08
Children	WAI	67	1.28	0.54	1	0	3	3	0.65	0.50	0.06			
	WNAI	45	0.89	1.05	0	0	3	3	0.69	−0.97	0.68	0.39	2.3	0.02
Km per week	WAI	67	107.4	112.73	80	2	500	498.00	1.57	1.83	14.43			
	WNAI	45	176.2	236.23	100	0	1000	1000.00	2.16	4.13	36.89	−68.8	−2	0.09

**Table 2:** Demographic variables and car crash history of the mothers attending infants (WAI) and not attending infants (WNAI).

		Group			
		WAI		WNAI	
		Count	Column N %	Count	Column N %
Currently employed	Yes	50	74.6%	36	80.0%
	Not	17	25.4%	9	20.0%
Coupled	Yes	63	94.0%	35	77.8%
	Not	4	6.0%	10	22.2%
Healthy	Yes	66	98.5%	45	100.0%
	Not	1	1.5%	0	0.0%
Time holding a driving licence	Less than 1 year	0	0.0%	1	2.2%
	Between 2 and 5 years	5	7.4%	4	8.9%
	More than 5 years	62	92.5%	40	88.9%
Currently driving	Yes	66	98.5%	39	86.7%
	Not	1	1.5%	6	13.3%
Have you ever had any driving incidents?	Yes	6	9.0%	10	23.3%
	Not	61	91.0%	33	76.7%
Severity of incident (if Yes answered above)	Slight	3	50.0%	7	70.0%
	Moderate	3	50.0%	2	20.0%
	Severe	0	0.0%	1	10.0%
Have you had any incidentssince your child was born?	Yes	4	6.0%		
	Not	63	94.0%		
Severity of the incident (if Yes answered above)	Slight	2	50.0%		
	Moderate	2	50.0%		
	Severe	0	0.0%		

### Fatigue and attention-related errors

Table 3 displays the descriptive statistics, the tests for the differences between means in the groups of mothers, and a measure of the consistency of the scales, Cronbach’s α, used in our study.

**Table 3:** Descriptive statistics by group for the chronic fatigue (FAS), acute fatigue (VAS FATIGUE), energy (VAS ENERGY) and attention-related errors when driving (ARDES) scales. The table also shows the results of Welch’s t-test for the groups, and Cronbach’s α. Mothers attending infants (WAI) and not attending infants (WNAI).

		N	Mean	St.Dev.	Median	Min	Max	Range	Skew	Kurt.	Std.Err.	dif	t	p	α
FAS	WAI	67	9.46	6.96	8.00	−2	29	31	0.88	0.29	0.85				
	WNAI	46	6.11	4.45	6.00	−2	21	23	0.78	1.06	0.66	3.35	3.1	0.00	0.86
VAS FATIGUE	WAI	67	49.84	22.99	46.00	14	102	88	0.47	−0.67	2.81				
	WNAI	46	34.50	20.07	27.50	13	98	85	1.39	1.33	2.96	15.33	3.7	0.00	0.93
VAS ENERGY	WAI	67	25.88	7.97	25.00	7	49	42	0.00	0.08	0.97				
	WNAI	46	31.98	7.57	32.00	17	45	28	−0.08	−0.93	1.12	−6.09	−4.1	0.00	0.95
ARDES	WAI	66	32.67	8.88	30.00	22	51	29	1.01	−0.163	1.09				
	WNAI	46	30.28	6.61	30.00	19	55	36	0.93	2.98	0.97	2.38	1.6	0.10	0.86

Mothers attending infants had a mean score of 9.45 in chronic fatigue—as measured by the FAS questionnaire—and women not attending them had a mean of 6.1. Differences between the two groups were significant (p < 0.01). Analogously, the two groups of women also manifested significant differences between fatigue at the moment of responding and its opposite (energy at the moment of responding). Thus, mothers attending or not infant children scored an average of 49.8 and 34.5 in the VAS FATIGUE scale (p < 0.01), and 25.88 and 31.98 in the VAS ENERGY scale (p < 0.01), respectively. All together, these results confirm the higher levels of fatigue−chronic and acute− and the lower level of energy of mothers attending infant children versus women not attending them.

Attention-related errors when driving, as measured by the scale ARDES, showed there were no significant differences between the means of the two groups of women (dif = 4.1, p < 0.05), which, in mediational analysis terminology, means no total impact of the independent variable on the dependent variable. However, note that the indirect effect can still be different from zero even if the total effect is not, as stated for example by^38^. Finally, all the scales exhibited satisfactory levels of internal consistency, with Cronbach’s alpha level ranging between 0.86 and 0.95.

Table 4 shows Pearson’s correlations between the variables in the study. Note that, as the group to which mothers belonged is included as a dummy variable, with the category of women not attending infants coded as one and the other group as zero, the correlations involving this variable are actually point-biserial correlations. Taking this into account, women not attending infants correlates negatively with chronic and acute fatigue (r = −0.26, p < 0.01; r = −0.33, p < 0.01), and positively with energy (r = 0.36, p < 0.01) and attentional driving errors−although the correlation is not significant (r = −0.15, p > 0.05). Chronic fatigue and acute fatigue are closely inter-correlated (r = 0.69, p < 0.01). Finally, chronic fatigue is positively related to attentional errors (r = 0.64, p < 0.01), just like acute fatigue (r = 0.47, p < 0.01). Finally, the correlations of ARDES with energy are moderate and negative, as expected (r = −0.30, p < 0.01).

**Table 4:** Correlations between women without infant children (WNAI), chronic fatigue (FAS), acute fatigue (VAS FATIGUE), and energy (VAS ENERGY). Note that **p < 0.01. α.

	WNAI	FAS	VAS FATIGUE	VAS ENERGY	ARDES
WNAI	1.00	−0.26**	−0.33**	0.36**	−0.15
FAS		1.00	0.69**	−0.48**	0.64**
VAS FATIGUE			1.00	−0.55**	0.47**
VAS ENERGY				1.00	−0.30**
ARDES					1.00

A visual inspection of the bivariate graphical displays of the variables did not provide evidence of the violation of the assumptions of linearity, homogeneity of variance or outliers.

### Mediation Analysis

**Figure 1:**
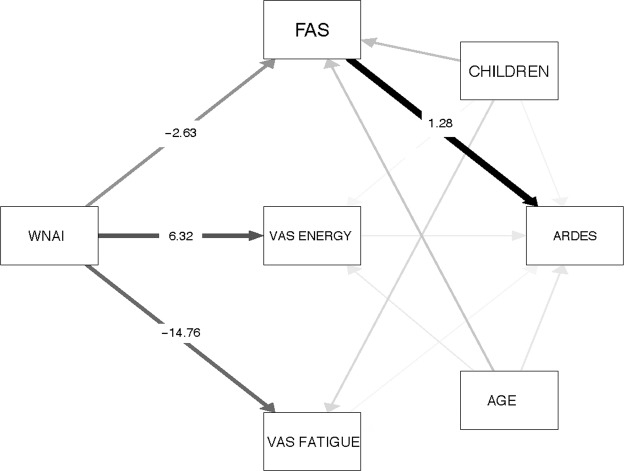
Mediated relationship between the group variable and attentional errors. The labels of the boxes are: WNAI = Women not attending infants; FAS = Chronic fatigue; VASF = Acute fatigue; VASE = Acute energy; ARDE = Attentional errors while driving, CHILDREN = Number of children of the woman, AGE = Age of the woman.

The hypothesis of the study stipulated that attending an infant child would increase fatigue in women, which, in turn, would predispose them to making attentional driving errors. This hypothesis was tested using a mediation model with the fatigue variables as mediators between the group variable (mothers attending infant children versus women not attending infant children) and the measurement of attentional errors. Additionally, two variables that it was thought might confound the results were also introduced into the analysis, namely: the age of the women participating in the study and the number of children. A graphical representation of this model is shown in Fig. 1. Note that the non-standardized coefficients of the model are displayed as numbers printed on the arrows, while the thickness of the edges is proportional to the standardized coefficients. In order to reduce clutter, only coefficients that reached statistical significance are displayed. As can be seen in the figure, the only indirect path connecting the group variable with the attentional errors has chronic fatigue as mediator. The confounding variables were not significantly related either to the mediator or the outcome variable.

Testing the difference of the coefficients from zero was carried out using 5,000 bootstrapped samples to estimate their intervals of confidence. The results are shown in Table 5 together with the Sobel test, the standardized coefficients (Beta), the non-standardized coefficients, and their standard errors.

**Table 5:** Mediated relationship between attending an infant child and attentional errors when driving. The labels of the variables stand for: Without = Women without infant children; FAS = Chronic fatigue; VAS FATIGUE = Acute fatigue; VAS ENERGY = Acute energy; ARDES = Attentional errors while driving.

Antecedent	Consequent	Coef.	s.e	z	p	Beta	CI Min	CI Max
WNAI	FAS (a1)	−2.62	1.12	−2.32	0.02	−0.21	−4.93	−0.28
WNAI	VAS_FATIGUE(a2)	−14.76	4.51	−3.27	0.00	−0.31	−22.9	−5.06
WNAI	VAS_ENERGY(a3)	6.32	1.55	4.08	0.00	0.37	3.16	9.29
FAS	ARDES (b1)	1.28	0.17	7.51	0.00	0.69	0.93	1.61
VAS FATIGUE	ARDES (b2)	−0.00	0.04	−0.18	0.85	−0.01	−0.01	0.08
VAS ENERGY	ARDES (b3)	−0.05	0.12	−0.42	0.67	−0.04	−0.28	0.19
WNAI-FAS-ARDES	in1 = (a1*b1)	−3.37	1.53	−2.20	0.02	−0.15	−6.81	−0.47
WNAI-VAS FATIGUE-ARDES	in2 = (a2*b2)	0.13	0.75	0.17	0.86	0.00	−1.28	1.75
WNAI-VAS ENERGY-ARDES	in3 = (a3*b3)	−0.32	0.79	−0.41	0.67	0.01	−1.92	1.26
WNAI (direct)	(c’)	0.76	1.72	0.44	0.65	0.03	−2.28	4.24
Total indirect effects	in1 + in2 + in3	−3.67	1.62	−2.20	0.02	−0.16	−7.29	−0.80
Total effect	(c)	−2.90	2.22	−1.30	0.19	−0.13	−7.31	1.56

The first part of Table 5 confirms our hypothesis that not currently attending an infant predicts more chronic and acute fatigue but less energy in the subjects, as the intervals of confidence do not include zero in any case. The second part of Table 5 shows that chronic fatigue predicts making attentional errors when driving, but acute fatigue and energy do not, when controlling for the effect of the other variables. The sign of the coefficients functions as expected too: not attending an infant child implies lower levels of chronic fatigue than attending one, and high levels of chronic fatigue imply a greater frequency of attentional errors.

The third part of Table 5 shows the estimates for the indirect effects of attending an infant child on attentional errors. As can be seen, the only one of them with a significant effect is that of attending an infant child on attentional errors mediated by chronic fatigue, as its bootstrapped interval of confidence does not include zero. On the other hand, the two indirect effects mediated by acute fatigue or energy are not significant. Finally, the direct relationship between not currently attending an infant child and the errors when driving is not significant, as the bootstrapped intervals of confidence do include zero.

## Discussion

As mentioned before, the academic literature on mothers attending infant children and driving pointed to the fact that this subpopulation should be considered as one at risk^6–9^. The results of this study concur with the previous literature in that this group of women has increased levels of fatigue, but also add that chronic fatigue predicts them having a higher frequency of attentional errors while driving. Attentional errors are caused by lapses in attention that occur when consciousness is absent or disengaged from current tasks^20^, a situation that seems quite common among women attending infants^7^ and which they describe as driving in automatic mode. All in all, this finding seems compatible with the theory that fatigue impairs adaptation to driving conditions, and as a consequence “complacency” problems may be increased^27^, i.e., fatigued drivers may try to make do without making sufficient cognitive effort, resorting to effortless automated behavior that may not suit the specific circumstances of the road.

Another finding of this study is that the measure of chronic fatigue acted as mediator variable for predicting attentional errors but the measures of acute fatigue did not. Of course, this result is partially a consequence of the close correlation between the different measures of fatigue, as in fact they were all individually good predictors of the errors. However, given the character of the measures, it seems fitting that the more general type of fatigue had more in common with a measure of errors that covered a wide range of situations and reached a longer time span than those limited to the specific moment of the test. Further research carried out in the laboratory might explore whether these measures would be useful for predicting the errors made throughout the experimental trial.

The current study presents several limitations that hint at future directions for research. First, note that the link between attentional errors and car crashes has not been tested: although it seems evident that cognitive lapses during driving might result in crashes, there is still the question of the degree to which the capacity of responding to unexpected events is absent in this population or, on the contrary, if the resources not yet worn off are sufficient for driving safely. Second, as the design used was a quasi-experiment^29^ in which the groups were not selected randomly, it is possible that the results found could be due to unaccounted for confounding variables—apart from the age of the mothers and the number of children. Third, the measures of fatigue and attention-related errors used were exclusively based on self-reports and, consequently, are prone to the biases often associated with these types of measures, such as wrong recalling, exaggerating, trying to confirm the experimenter’s perceived hypothesis, and so forth. Therefore, it seems appropriate to confirm the findings of this study using more objective measures taken in the laboratory using a driving simulator, or in real driving conditions.
